# Decontaminative Properties of Cold Atmospheric Plasma Treatment on Collagen Membranes Used for Guided Bone Regeneration

**DOI:** 10.3390/jfb14070372

**Published:** 2023-07-14

**Authors:** Aydin Gülses, Lina Dohrmann, Oral Cenk Aktas, Juliane Wagner, Salih Veziroglu, Tim Tjardts, Torge Hartig, Kim Rouven Liedtke, Jörg Wiltfang, Yahya Acil, Christian Flörke

**Affiliations:** 1 Department of Oral and Maxillofacial Surgery, University Hospital Schleswig-Holstein, 24105 Kiel, Germanyjuliane.wagner@uksh.de (J.W.); joerg.wiltfang@uksh.de (J.W.); yahya.acil@uksh.de (Y.A.); christian.floerke@uksh.de (C.F.); 2 Chair for Multicomponent Materials, Institute for Materials Science, Faculty of Engineering, Christian-Albrechts-University of Kiel, 24118 Kiel, Germany; oca@tf.uni-kiel.de (O.C.A.); sve@tf.uni-kiel.de (S.V.); tt@tf.uni-kiel.de (T.T.); toha@tf.uni-kiel.de (T.H.); 3 Kiel Nano, Surface and Interface Science KiNSIS, Kiel University, Christian Albrechts-Platz 4, 24118 Kiel, Germany; 4 Department of Orthopedics, University Hospital Schleswig-Holstein, 24105 Kiel, Germany

**Keywords:** carbon, cold atmospheric plasma, *E. faecalis*, collagen, guided bone regeneration

## Abstract

Background cold atmospheric plasma (CAP) is known to be a surface-friendly yet antimicrobial and activating process for surfaces such as titanium. The aim of the present study was to describe the decontaminating effects of CAP on contaminated collagen membranes and their influence on the properties of this biomaterial in vitro. Material and Methods: A total of *n* = 18 Bio-Gide^®^ (Geistlich Biomaterials, Baden-Baden, Germany) membranes were examined. The intervention group was divided as follows: *n* = 6 membranes were treated for one minute, and *n* = 6 membranes were treated for five minutes with CAP using kINPen^®^ MED (neoplas tools GmbH, Greifswald, Germany) with an output of 5 W, respectively. A non-CAP-treated group (*n* = 6) served as the control. The topographic alterations were evaluated via X-ray photoelectron spectroscopy (XPS) and scanning electron microscopy (SEM). Afterward, the samples were contaminated with *E. faecalis* for 6 days, and colony-forming unit (CFU) counts and additional SEM analyses were performed. The CFUs increased with CAP treatment time in our analyses, but SEM showed that the surface of the membranes was essentially free from bacteria. However, the deeper layers showed remaining microbial conglomerates. Furthermore, we showed, via XPS analysis, that increasing the CAP time significantly enhances the carbon (carbonyl group) concentration, which also correlates negatively with the decontaminating effects of CAP. Conclusions: Reactive carbonyl groups offer a potential mechanism for inhibiting the growth of *E. faecalis* on collagen membranes after cold atmospheric plasma treatment.

## 1. Introduction

Collagen is a common biomaterial in regenerative medicine due to its great biocompatibility, low antigenicity, prominent cell affinity, and biodegradability that has the potential to repair tissues and restore their physiological function [1,2,3]. Decellularized biodegradable collagen membranes are frequently utilized in dentistry to generate various compartments for the repair of critical size defects. Collagen membranes are used in both guided bone regeneration and guided tissue regeneration to act as an occlusive barrier to stop gingival soft tissue from growing into a periodontal or bone defect. This enables tissue regeneration through the unrestricted proliferation and differentiation of site-specific progenitor cells.

A bilayer membrane called Bio-Gide^®^ is intended to protect a bone defect from soft tissue invasion and has been used in dental medicine for several decades [4] in the management of periodontal and peri-implant diseases or to enhance the ossification of bone defects of any origin. In order to allow for bone ingrowth, Bio-Gide^®^’s rough side must face the bone defect, whereas the smooth layer should bear the soft tissues.

The width, spacing, and orientation of membrane fibers or fibrils, as well as the stiffness of the membrane, have all been found to influence cellular behaviors like differentiation, migration, and proliferation [5,6,7,8,9]. However, membrane exposure, which leads to bacterial contamination and often results in the disintegration of the graft, has been reported to be the most common complication secondary to guided bone/tissue regeneration [10], which mostly necessitates the complete removal of the biomaterials placed [11]. Additionally, it has been reported that membrane exposure after GBR procedures has a significant detrimental influence on the outcome of bone augmentation. Garcia et al. [12] have reported that the sites without membrane exposure achieved 74% more horizontal bone gain than the sites with exposure.

The functionalization of collagen-based biomaterials aims to modify their chemical and biological characteristics and helps to develop clinical strategies against bacterial contamination without impairing the regenerative properties. Taraballi et al. [13] enriched amine and carboxylic functional groups on collagen films by using N_2_/H_2_ and CO_2_ plasma and investigated the physico-chemical features of the modified surfaces as well as their in vitro biocompatibility in the presence of human osteoblast-like cells. Eggers et al. [14] also showed that plasma treatment significantly improved osteoblast-like cell viability when compared to untreated cells and proclaimed that plasma treatment has a positive impact on wound closure in an in vitro setting.

In addition to its regenerative effects, numerous investigations [14,15,16,17,18] conducted over the past 20 years have also established the antibacterial activity of plasma treatment. It has been proclaimed that cold atmospheric plasma (CAP) has the capacity to enter porous surfaces, efficiently disinfecting the intricate three-dimensional surface [19]. It was also proven that CAP might inactivate biofilm on micro-structured titanium surfaces without altering its topography. These studies also demonstrated that plasma could reduce the number of living bacteria on the micro-structured surface of dental titanium implants. However, the effects of CAP on different biomaterials remain unclear. Additionally, the duration of treatment is another point that has been discussed controversially in the literature [20,21].

It is well known that free radicals are a source of oxidative stress and are hostile to bacteria [20,22]. Many studies have demonstrated that an increased intracellular level, which is caused by a direct CAP jet, can either react primarily with the cell envelope or damage intracellular components and subsequently lead to bacterial death [23]. Most of these studies have exposed the bacterial suspension directly under the CAPs, with the bacterial growth being influenced by the forthright oxidative stress caused by free radicals [23]. However, Yang et al. [21] recently showed that the free radicals left on the zirconia surface can impose oxidative stress on bacteria after CAP treatment. Therefore, it is important to investigate the mechanisms of bacterial death on the CAP-treated materials to encourage or avoid the use of CAP as a promising surface modification method in clinical implant dentistry. The effects of CAP on collagen membranes have not been evaluated until now. The present study’s objective was to describe the decontaminative effects of CAP on contaminated collagen membranes and its influence on surface characteristics in vitro.

## 2. Materials and Methods

### 2.1. Membranes

Bio-Gide^®^ (Geistlich Biomaterials, Baden-Baden, Germany), which acts as a bilayer barrier made of porcine dermis type I and III collagen, was selected. The membranes were cut in 10×10 mm samples. A total of 18 samples were used.

### 2.2. CAP Treatment

**Figure 1:**
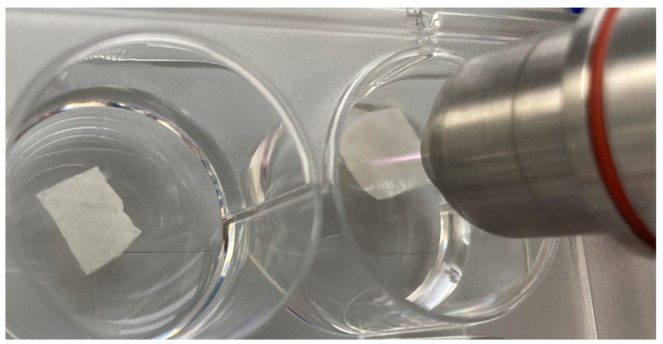
Treatment of collagen membrane with cold atmospheric plasma using kINPen^®^ MED.

The rough surface of each membrane in the first group (*n* = 6) was treated with cold plasma for 1 min. In the second group (*n* = 6), each membrane was treated for 5 min by also using kINPen^®^ MED (neoplas tools GmbH, Greifswald, Germany) with an output of 5 W, respectively (Figure 1). A non-CAP-treated group (*n* = 6) served as the control.

### 2.3. Surface Characterization

For surface characterization, non-contaminated membranes were CAP treated for 1 min and 5 min, respectively. A group containing non-CAP-treated membranes served as the control sample. To emulate the possible changes regarding cultivation, as described below, the membranes were cultivated in 10 mL of sterile nutrient solution, which was exchanged every 24 h for 6 days. The surface morphology of the membranes was revealed by scanning electron microscopy (SEM; Supra55VP-Carl Zeiss, Oberkochen, Germany). Furthermore, X-ray photoelectron spectroscopy (XPS; Al anode, 240 W Omicron Nano-Technology GmbH, Taunusstein, Germany) measurements were performed to determine the chemical states on the membrane surface. All the binding energies were referenced to the C 1s peak at 285.0  eV of the adventitious carbon on the surface.

### 2.4. Bacterial Contamination

Bacterial contamination was performed as previously described by Flörke et al. [24]. Briefly, immediately after the CAP treatment, cultivation with 10 mL of sterile nutrient solution (BHI, Brain–Heart-Infusion Broth, Carl Roth GmbH + Co. KG, Karlsruhe, Germany) and 100 μL of bacterial culture with *E. faecalis* (ATCC 29,212) was conducted at 37 °C for 24 h (Heraeus B6060, Heraeus Holding GmbH, Hanau, Germany). Afterward, the boxes (Eppendorf pipette tip reusable boxes Eppendorf AG, Hamburg, Germany) containing the models were sterilized at 121 °C (Autoclave Melag Vacuklav 24, MELAG Medizintechnik oHG, Berlin, Germany). On the first day, the membranes were infected with 200 mL of sterile BHI and 100 μL of the overnight culture and then incubated at 37 °C (Scientific C24 Incubator Shaker, New Brunswick Scientific, Edison, NJ, USA). After 4 h, the optical density was controlled via a spectrophotometer (BioPhotometer 6131, Eppendorf AG, Hamburg, Germany) at 600 nm (OD600), which was set to 0.8. The nutrient solution was exchanged every 24 h with 200 mL of sterile BHI for 6 days.

### 2.5. Evaluation of the Bacterial Decontamination

The bacterial decontamination of the samples was quantified by counting the colony-forming units (CFUs) and qualified by using a scanning electron microscope, as previously described by Flörke et al. [24].

#### 2.5.1. Colony-Forming Units

Each membrane was placed in an Eppendorf tube containing 1 mL of sterile NaCl solution. To de-attach the bacteria from the surface, the membranes were placed in an ultrasonic bath (ultrasonic bath Branson 2210R-MT Ultrasonic Cleaner, Branson Ultrasonics Corporation, Danbury/CT, USA) for 20 min. The bacterial suspension was then diluted to 10–2 and later to 10–4. Afterward, the different dilution levels were applied on Caso agar plates. These Caso agar plates (CASO-Agar Ph.Eur., Carl Roth GmbH + Co. KG, Karlsruhe, Germany) were placed in an incubator at 37 °C (Heraeus B6060 incubator, Heraeus Holding GmbH, Hanau, Germany). After 24 h, the colony-forming units were counted with a germ counter (Germ counter BZG 25 from WTW, Xylem Analytics Germany Sales GmbH & Co. KG, Weilheim, Germany).

#### 2.5.2. Scanning Electron Microscope

The membranes were washed for 1 min with PBS (phosphate-buffered saline solution, Dulbeco, Biochrom GmbH, Berlin, Germany) and later with 1 mL of 4% glutaraldehyde. Afterward, the membranes were washed three times for 5 min, each time with PBS. The dehydration was carried out by means of an ascending series of 30%, 50%, 70%, 90%, and 100% ethanol. The membranes were then air-dried until the ethanol was completely evaporated. The membranes were then attached to SEM sample plates (Agar Scientific Ltd., Stansted, Essex, UK) and stored overnight in a desiccator (Erich Eydam KG, Kiel, Germany). Gold sputtering at a thickness of 5 nm (BAL-TEC SCD 500, Leica Microsystems GmbH, Wetzlar, Germany) and examination using a scanning electron microscope (Philips XL 30 ESEM, Philips GmbH Market DACH, Hamburg, Germany) was performed. For each membrane, five corresponding areas at a magnification of 5000× and five areas at a magnification of 8000× were recorded.

### 2.6. Statistical Analysis

Descriptive statistical analysis was carried out using IBM, SPSS, Statistics version 24.0 for Windows (IBM GmbH, Ehningen, Germany). A paired *t*-test was conducted to evaluate the statistical differences between the groups (non-treated vs. CAP for 1 min. and non-treated vs. CAP for 5 min). The significance level was set to (*p* < 0.05).

## 3. Results

### 3.1. SEM Analysis of the Surfaces

**Figure 2:**
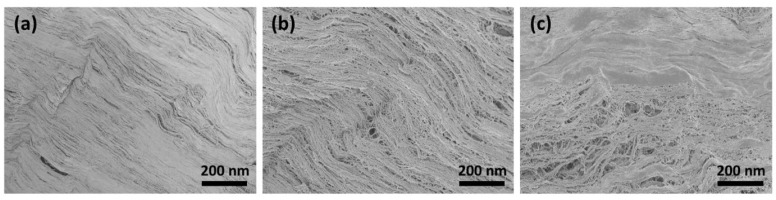
SEM image of (**a**) untreated collagen membrane and following CAP treatment for (**b**) 1 min and (**c**) 5 min.

The SEM images show that the surface morphology changed fundamentally after treatment with CAP. Figure 2a shows the smooth surface morphology of the untreated collagen membrane. It can be clearly seen from Figure 2b that the membrane surface still had many collagen fibrils that were closely intertwined after CAP treatment for 1 min. These fibril bundles reveal a typical periodic banding pattern. However, following CAP treatment for 5 min, the caudal side clearly shows circular irregularities and discontinuities (Figure 2c). Here, CAP might chemically eliminate/degrade the materials in those regions.

### 3.2. XPS Analysis

**Figure 3:**
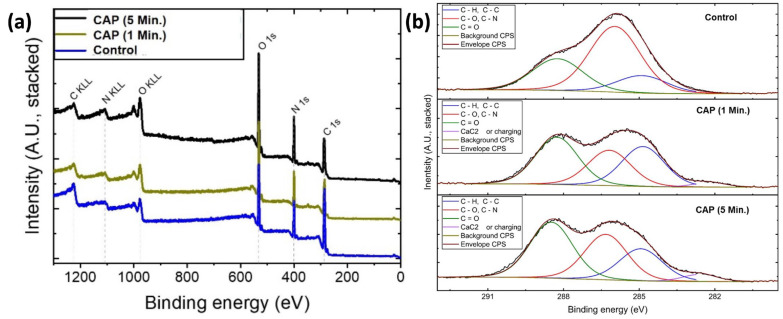
(**a**) Wide scan and (**b**) high-resolution carbon XPS spectra of untreated and treated samples for different time intervals.

In order to identify the elemental composition and chemical state of the membrane surface, XPS spectra of the samples (before and after CAP treatment) were recorded, as shown in Figure 3. As expected for the collagen membrane, the wide-scan XPS spectrum showed the presence of carbon (C), nitrogen (N), and oxygen (O) on the sample surface without any external contamination (Figure 3a). Additionally, the XPS survey shows that after CAP treatment, no major contaminations from the preparation and processing steps were observed (Figure 3a). However, increasing CAP time significantly enhanced the carbon concentration from 27.68% to 40.39% (carbonyl group: C=O; corresponding peak position around 288.5 eV) on the surface (Figure 3b). This might be explained by the fact that CAP can start degrading the organic materials in the fibrils, which causes a significant increase in the carbon content on the surface. Besides, the incorporation of Argon due to the plasma pen is negligible and was not observed.

The relative distribution of carbon functional groups from integration and the relative and absolute intensities of the carbon functional groups representing the fitting functions are shown in Table 1. The peak intensity was determined as the area between the peak and the baseline.

### 3.3. Colony-Forming Units

**Figure 4:**
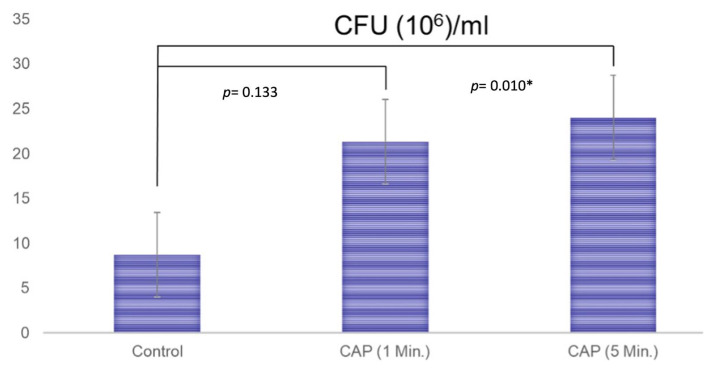
The results of the formation of colony-forming units for the untreated membranes (Control; 8.7 ± 11.2), the membranes that were treated with CAP for 1 min (21.3 ± 8.6), and the membranes that were treated with CAP for 5 min (24.0 ± 4.4). A paired *t*-test was performed to test for significance. A significant difference was detected between untreated and CAP-treated membranes when treatment was performed for 5 min (*p* = 0.010). * showing the statistically significance.

The results of the paired-t test indicated that there is a difference between the non-treated (8.7 ± 11.2) and CAP-treated (21.3 ± 8.6) membranes (treatment for 1 min) regarding the CFU values (*p* = 0.133), but even if this medium difference is present, it fell short of the significance level. After CAP treatment for 5 min, a significant difference between the formation of the CFUs (24.0 ± 4.4) could be detected (*p* = 0.010, Figure 4).

### 3.4. SEM Analysis

**Figure 5:**
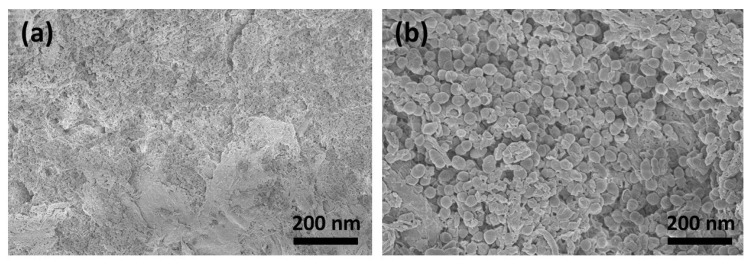
Six days after contamination, the surface (**a**) was fully covered with micro-organisms and conglomerates (**b**).

**Figure 6:**
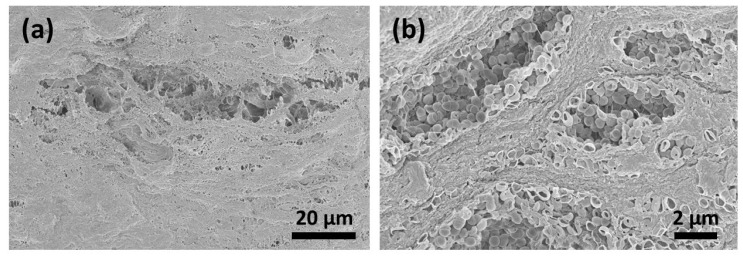
CAP treatment for 1 min resulted in a decrease in bacterial colonization on the surface of the collagen material. The destruction of the bacterial membranes could be detected (**a**). In the deeper layers, the bacterial conglomerates were still remarkable (**b**).

**Figure 7:**
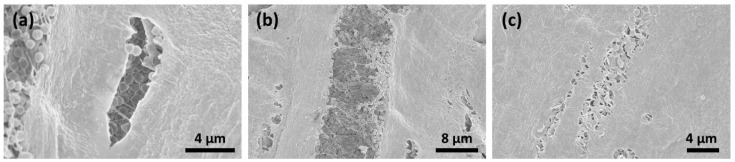
SEM analysis after CAP treatment for 5 min resulted in the decontamination of the membrane surface; however, bacterial conglomerates remained in the deeper layers (**a**–**c**).

Six days after contamination, many micro-organisms and conglomerates on the surfaces could be seen (Figure 5a,b). After CAP treatment for 1 (Figure 6a,b) and 5 (Figure 7a–c) minutes, the surface of the membranes was free from bacteria; however, the deeper layers showed the remaining conglomerates.

## 4. Discussion

Within the last two decades, the plasma treatment of dental biomaterials has become the main subject of many studies. The majority of the CAP strategies are based on the management of the improvement of cell adhesion on titan and zirconia surfaces and are aimed at optimizing the osseointegration process [25]. On the other hand, CAP treatment has also been shown to be an efficient method for the management of peri-implantitis when combined with mechanical debridement [24], thanks to its antimicrobial effects [26].

Stimulatory effects and CAP-induced cell mobility seem to play a major role in higher cell viability and improved cell migration [27,28]. On the other hand, it is very well known that CAP treatment can improve the surface wettability of biomaterials [29]. Duske et al. [30] showed that CAP treatment reduces the contact angle and supports the spreading of osteoblastic cells and suggested that the application of cold plasma may be supportive in the treatment of peri-implant lesions and may improve the process of re-osseointegration. Similarly, Wagner et al. [24] proclaimed that the healing capacity provided through CAP treatment could enhance the osseointegration of dental implants and has the potential to serve as an effective treatment option in periimplantitis therapy. However, it is very well documented that surfaces with a higher wettability show higher bacterial adhesion and/or bacterial colonization [31]. Therefore, despite its antibacterial effects, alterations to surface topography were correlated with bacterial adhesion. The aim of peri-implantitis therapy is to decontaminate the implant surface and, if possible, induce the regeneration of the peri-implanted tissues. For this reason, it might be speculated that there exists a danger in proposing CAP in the management of peri-implantitis due to topographical alterations after CAP treatment and the possible influence of this on bacterial adhesion.

Membrane exposure, following guided bone/tissue regeneration, presents a great challenge for the dental clinician; thus, bacterial contamination can lead to an infection and, therefore, necessitates the complete removal of any placed biomaterials. In recent years, novel strategies to enhance the antimicrobial effect have been proposed by changing the membrane surface or incorporating long-term released antimicrobials [32]. The current ex vivo study aimed to answer a clinical question based on the following hypothesis: can CAP treatment allow for bacterial decontamination in the case of membrane exposure after guided bone/tissue regeneration? Despite its limitations, such as the use of an ex-vivo experimental model with mono-bacterial decontamination, the current study showed that CAP treatment could reduce bacterial colonization only on the surface of the collagen membrane. Despite successful decontamination of the surface of the membrane, quantification via colony-forming unit counts showed an increase after CAP treatment, which also correlates significantly with the duration of CAP application. This effect might be attributed to the alterations to the topography and the increased carbonyl content of the material after CAP treatment.

From the perspective of the materials sciences, plasma treatment is often used to modify the surface properties of polymer films since it offers numerous advantages over conventional surface modification techniques [33]. Azam et al. stated that the availability of functional groups (N–H and C–H) might promote adhesion on dental biomaterials after plasma treatment [34]. Similarly, Morent et al. showed that plasma treatment leads to the incorporation of C–O, C=O, and O–C=O groups on polymers [35]. In a recent article, Yang et al. demonstrated that reactive oxygen species offer a potential mechanism for inhibiting the growth of *S. mutans* on zirconia surfaces treated with cold atmospheric plasma. The increase in bacterial proliferation in the CAP-treated groups verified by CFU assays in our study could be explained by the fact that surface carbonyl (C=O) groups tend to promote bacterial growth and activity.

## 5. Conclusions

Cold atmospheric plasma fundamentally changes the morphology of collagen membranes, and bacterial colonization could be physically eliminated from the surface. However, the deeper layers of the membranes, especially, do not undergo decontamination at all and seem to build a microbial reservoir. Therefore, the decontamination of collagen membrane surfaces using a plasma pen might not be an option in the management of exposed membranes that are used for guided bone/tissue regeneration.

## Figures and Tables

**Table 1:** Relative distribution of carbon functional groups from integration.

	C–H, C–C/at%	C–O, C–N/at%	C=O/at%	CaC_2_ or Charging
Untreated	15.18	57.14	27.68	NaN
5 min	31.67	28.12	37.67	2.53
1 min	23.02	32.71	40.39	3.88

## Data Availability

The datasets used and/or analyzed during the current study are available from the author (yahya.acil@uksh.de) upon reasonable request.
